# Risk factors, diagnosis, and long-term erectile dysfunction outcomes in priapism: a retrospective analysis of 186 cases from a single institution

**DOI:** 10.1038/s41443-025-01076-9

**Published:** 2025-04-22

**Authors:** Joseph A. Borrell, Anthony Bettencourt, Thiago P. Furtado, Catherine Gu, Nancy Ye, Juan J. Andino, Sriram V. Eleswarapu, Jesse N. Mills

**Affiliations:** 1 David Geffen School of Medicine, University of California, Los Angeles, CA USA; 2 Department of Urology, University of California, Los Angeles, CA USA

**Keywords:** Risk factors, Quality of life

## Abstract

A retrospective analysis of 186 priapism cases between 2015–2024 was conducted using ICD-10 codes (N48.3) in a single high-volume institution. Data on age, race, insurance status, priapism subtype, etiology, and treatment outcomes were collected. The median age at presentation was 50.5 years (IQR: 39.3–60.0), with most patients being White (60.2%). Black and Asian patients tended to present at younger ages than White patients (45 and 38 vs. 55.5 years, *p* < 0.05). Black patients had longer priapism durations than White patients (36 vs. 7.8 h, *p* < 0.001). Commercial insurance was most common (60.2%), but those with Medi-Cal/Medicaid or self-pay had longer episodes compared to commercial payers (25 and 39 vs. 7 h, respectively, *p* < 0.05). Acute ischemic priapism was the most common subtype (63.4%), with intracavernosal injection therapy (54.8%) as the leading cause followed by medications such as Trazodone. Multivariate analysis revealed that ischemic priapism duration was the strongest predictor of de novo ED, with episodes lasting more than 36 h significantly increasing the risk (OR = 61.3, *p* < 0.001), although episodes over 20 h were also found to increase the risk (OR = 25.2, *p* = 0.007). These results emphasize the importance of early intervention and addressing health disparities to reduce long-term complications.

## Introduction

Priapism is a medical condition defined by a prolonged, usually painful, erection that may lead to corporal fibrosis and subsequent erectile dysfunction (ED) if left untreated [1]. Priapism is often classified into three main subtypes: nonischemic (high-flow), ischemic (low-flow), and recurrent. Each subtype has its own unique pathophysiological mechanism, clinical presentation, and treatment approach. While a rare urologic condition, the incidence of priapism is thought to account for 8 out of every 100,000 emergency department visits [2], although the exact incidence remains unknown.

Priapism has been recognized for centuries, yet despite the existence of updated American Urological Association (AUA) guidelines [1], current clinical experience with the condition remains inadequately documented due to its rarity. Known risk factors for priapism include hematological disorders such as sickle cell disease [3], medication use (particularly antidepressants and antipsychotics) [4], trauma [5], and illicit drug use [6, 7]. Recent literature also indicates that ischemic priapism may be increasingly prevalent in patients utilizing intracavernosal injection (ICI) therapy for ED [8]. Additionally, there is emerging evidence suggesting an increasing incidence of priapism, particularly among high-risk populations such as those with sickle-cell disease [9].

Priapism often requires a multidisciplinary approach, including the emergency department, urology, and a primary care provider (PCP). Therefore, this study aims to review the contemporary incidence of priapism, examining epidemiological trends and contributing risk factors to provide a clearer understanding of priapism, profiling diagnostic approaches, management, and ED outcomes. To our knowledge, this is the one of the largest cohorts of priapism cases specifically analyzed for socioeconomic and long-term ED outcomes.

## Methods

### Identification and data collection

We identified all patients at a single high-volume institution from November 2015 to September 2024 with ICD-10 codes for Priapism (N 48.30, N48.31, N48.32, N48.33, N48.39). Emergency department visits, urgent care visits, outpatient clinic visits, and inpatient cases were all included in the analysis. Inclusion criteria encompassed those who presented with true nonischemic, acute ischemic, or recurrent ischemic priapism. Patients who initially presented at an outside hospital (OSH) and then transferred to our institution were only included if medical records from the OSH were present in the electronic medical record (EMR). Exclusion criteria included those with incomplete medical record documentation, priapism outside the time frame, unspecified penile pain and erections, painful nocturnal erections, and penile pain associated with other disorders, including but not limited to Peyronie’s disease, infection, phimosis, and chordee.

Two independent urology researchers performed a retrospective chart review on all patients (JB and AB). Information on the following was extracted: date of presentation, age at presentation, race/ethnicity, location of presentation (emergency department, outpatient clinic, urgent care, or inpatient admission), insurance status, comorbidities, hematologic conditions, duration of priapism, etiology, type of priapism, psychiatric medication(s) at presentation, diagnostics, treatment (medical, procedural, and surgical), and ED status and therapy before and after priapism.

### Subtype and etiological classification

Priapism classification followed the 2022 AUA guidelines [1]. Acute ischemic priapism included those patients with a single priapism episode, while recurrent priapism included those with multiple episodes of acute ischemic priapism with interval periods of detumescence. For those classified as recurrent priapism, analysis was performed only on the first presentation of priapism within the time frame. Non-ischemic priapism was identified by a corporal blood gas (CBG) indicating arterial flow. If no blood gas was obtained but an intervention occurred, classification was based on expert consensus from the two most senior faculty authors. The study also included patients with prolonged erections of <4 h if clinical judgment required intervention. Regarding etiology, patients without a provider-listed etiology in the EMR were classified as “idiopathic,” even if they may have been taking high-risk medications or using illicit drugs (cocaine, marijuana, methamphetamine, benzodiazepine, or MDMA) at presentation.

### Statistical analysis

Categorical data on demographics, etiology, subtype, ED outcomes, and treatment were analyzed using chi-square and Fisher’s exact tests. Median ages and durations were compared using the Mann-Whitney U test. A point-biserial correlation assessed the relationship between priapism duration and follow-up status. Multivariate analysis evaluated the impact of ischemic priapism duration, age, and comorbidities on ED outcomes, with patients grouped by duration (0–4, 4–8, 8–12, 12–20, 20–36, 36+ hours). The 0–4 h group served as the reference for ED risk, assuming this group had the lowest likelihood of developing ED. All analyses were performed in Excel and R (Version 4.4.1), with statistical significance defined as *p* < 0.05.

## Results

**Fig. 1:**
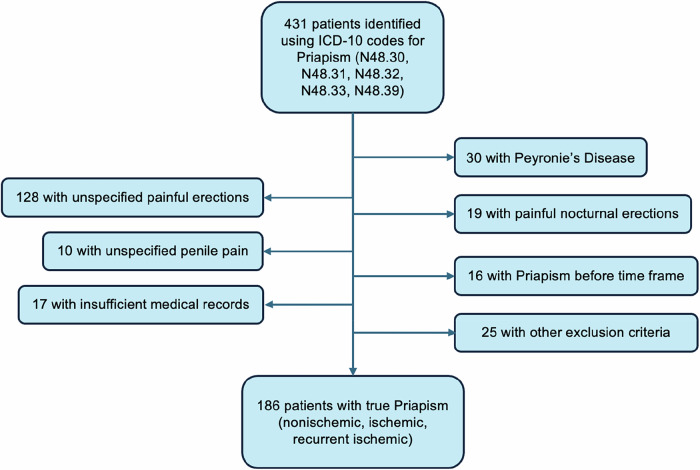
Priapism identification, inclusion, and exclusion approach.

Using ICD-10 codes for priapism (N48.3), 431 unique patients were identified. Of those, 186 (43.2%) met inclusion criteria and made up our analysis. (Fig. 1).

Demographic, comorbidity, and insurance data on the 186 true priapism cases are listed in Table 1. The median age at presentation was 50.5 years (IQR: 39.3–60.0). The majority of patients were White (60.2%), followed by Black/African American (15.6%), and Asian (4.8%). 10.2% of patients identified as Hispanic/Latino. 14.0% had a diagnosis of diabetes, 41.4% with hypertension, and 27.4% with hyperlipidemia. 40.9% had pre-existing ED before their initial presentation with priapism. Regarding hematological comorbidity, 4.3% had documented sickle cell disease/trait, 5.4% had active cancer at presentation, and 1.6% had other hematological conditions, including but not limited to thalassemia (minor or major), hemochromatosis, or polycythemia (polycythemia vera or secondary polycythemia). 11.8% had Human Immunodeficiency Virus (HIV).

**Table 1:** Demographic and comorbidity information at initial presentation with priapism.

Median Age at Presentation (IQR)	50.5 years (39.3–60.0)
Race (%)	
White	60.2
Black or African American	15.6
Asian	4.8
American Indian, Alaska Native, Pacific Islander	1.6
Other Race	7.0
Unknown/Declined to Answer	10.8
Ethnicity (%)	
Not Hispanic/Latino	78.0
Hispanic/Latino	10.2
Unknown/Declined to Answer	11.8
Comorbidities (%)	
Diabetes	14.0
Hypertension	41.4
Hyperlipidemia	27.4
Erectile Dysfunction	40.9
Active Cancer (any)	5.4
Sickle Cell Disease/Trait	4.3
Other Hematologic Conditions (Thalassemia, Polycythemia, Hemochromatosis)	1.6
Systemic Lupus Erythematosus (SLE)	1.1
Human Immunodeficiency Virus (HIV)	11.8
Insurance Status (%)	
Commercial	60.2
Medi-Cal/Medicaid	19.9
Medicare	17.7
Self-Pay	2.2
Location of Initial Presentation (%)	
Emergency Department	70.4
Outpatient Clinic (Urology Provider or Primary Care Provider)	26.9
Urgent Care	0.5
Inpatient Admission	2.2

Regarding the median age at presentation, Black/African American and Asian patients were significantly younger than their White counterparts, with median ages of 45 years and 38 years, respectively, compared to 55.5 years for White patients (*p* < 0.05) (Table 2). Additionally, Black/African American patients were more likely to present with longer-duration priapism than White patients (*p* < 0.001) (Table 2). No significant differences in median age or priapism duration were observed between Hispanic/Latino and non-Hispanic/Latino patients.

**Table 2:** Median ages and priapism durations for race, ethnicity, and insurance status.

	Median Age at Presentation, years (IQR)	*p*-value (Age)	Median Duration of Priapism, Hours (IQR)	*p*-value (Duration)
Race				
White (*n* = 112)	55.5 (42–62)	*Reference*	7.8 (4–24)	*Reference*
Black/African American (*n* = 29)	45 (37–53)	**0.004**	36 (10–60)	**0.001**
Asian (*n* = 9)	38 (29–43)	**0.002**	10 (4–10)	0.503
American Indian/Pacific-Islander (*n* = 3)	59 (41–64)	0.909	6 (5.5–51)	0.798
Ethnicity				
Non-Hispanic/Latino (*n* = 145)	51 (39–61)	*Reference*	9 (4–33)	*Reference*
Hispanic/Latino (*n* = 19)	48 (30–54)	0.103	14 (5–30)	0.545
Insurance Status				
Commercial (*n* = 112)	51 (43–59)	*Reference*	7 (4–17)	*Reference*
Medi-Cal/Medicaid (*n* = 37)	40 (29–46)	**<0.001**	25 (10–51.5)	**<0.001**
Medicare (*n* = 33)	66 (42–68)	**<0.001**	7 (4–24)	0.878
Self-pay (*n* = 4)	49 (44.5–53.2)	0.639	39 (25.8–48)	**0.041**

**Statistical Analysis:** Median ages and durations were compared using the Mann-Whitney U test. Fisher’s exact test was applied for categorical variables with counts less than five. A *p*-value of <0.05 was considered statistically significant.

Regarding insurance status, patients with Medi-Cal/Medicaid were younger and presented with significantly longer-duration priapism compared to those with commercial insurance (*p* < 0.001). Similarly, self-pay patients also tended to present with longer-duration priapism compared to those with commercial insurance (*p* < 0.001; Table 2).

**Fig. 2:**
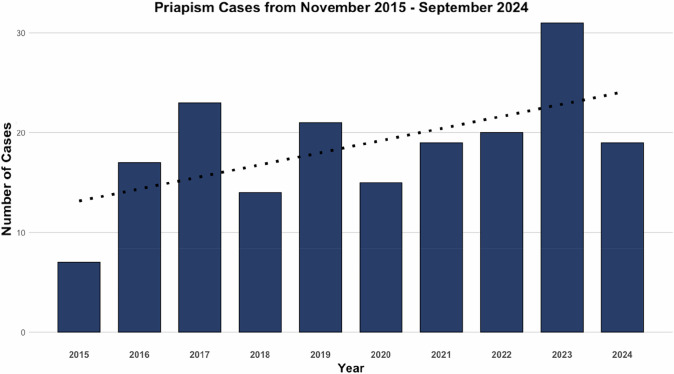
Unique priapism cases from November 2015 to September 2024, including emergency department and outpatient clinic visits.

The majority of the patients (70.4%) initially presented to the emergency department, with 26.9% presenting to an outpatient clinic (Urology clinic or a PCP). There was a noted trend of increasing priapism cases during the study period (Fig. 2).

The majority of priapism cases (63.4%) were classified as acute ischemic priapism, while 32.8% were identified as recurrent ischemic and 3.8% as nonischemic (Table 3). For those with recurrent priapism, there was an average of 1.72 ± 2.61 additional episodes of priapism.

**Table 3:** Etiologies of priapism with associated median durations and priapism subtype classification.

Etiology	Median Duration of Priapism, Hours (IQR)	Acute Ischemic, n (%)	Recurrent Ischemic, n (%)	Nonischemic, n (%)
***Idiopathic (n**** = ****28)***	24 (10–70.5)	12 (42.9)	16 (57.1)	0 (0)
***Intracavernosal injection (ICI) therapy (n**** = ****102)***	6 (4–12)	77 (75.4)	25 (24.5)	0 (0)
*Provider-administered (n = 40)*	3 (2–4)	39 (97.5)	1 (2.5)	0 (0)
*Self-administered, prescribed (n = 40)*	7 (6–12)	23 (57.5)	17 (42.5)	0 (0)
*Self-administered, recreational (n = 22)*	30 (12–63)	15 (68.2)	7 (31.8)	0 (0)
**Medications (*n*** = **42)**	11 (7–48)	24 (57.1)	15 (35.7)	3 (7.1)
*Trazodone* (*n* = 27)	24 (7.5–48)	15 (55.6)	10 (37.0)	2 (7.4)
*Quetiapine* (*n* = 5)	8 (6–10)	2 (40.0)	2 (40.0)	1 (20.0)
*Escitalopram* (*n* = 1)	7 (7–7)	0 (0)	1 (100.0)	0 (0)
*Sildenafil* (*n* = 2)	29.5 (20.2–38.8)	1 (50.0)	1 (50.0)	0 (0)
*Tadalafil* (*n* = 2)	13.5 (10.2–16.8)	1 (50.0)	1 (50.0)	0 (0)
*Tamsulosin* (*n* = 2)	5.5 (5.3–5.8)	2 (100.0)	0 (0)	0 (0)
*Other* (*n* = 3)	**N/A**^a^	3 (100.0)	0 (0)	0 (0)
**Sickle Cell Disease/Trait (*n*** = **7)**	24 (17–480)	3 (42.9)	4 (57.1)	0 (0)
**Illicit Drug Use (*n*** = **3)**	144 (90–156)	2 (66.7)	1 (33.3)	0 (0)
**Trauma (*n*** = **4)**	**N/A**^a^	0 (0)	0 (0)	4 (100.0)
Total	9.5 (4–36)	118 (63.4)	61 (32.8)	7 (3.8)

^a^*N/A* not available.

Overall, the median priapism duration was 9.5 h (IQR: 4.0–36.0) (Table 3). ICI therapy was the most common etiology, accounting for 54.8% of cases, with recreational ICI users having longer-duration priapism compared to prescribed or provider-administered ICI users (*p* < 0.05). Medication-induced and idiopathic priapism followed, representing 22.5 and 15.1% of cases, respectively. Trazodone was the most common medication linked to priapism, accounting for 64.3% of medication-induced cases and 14.5% overall, while quetiapine accounted for 11.9% of medication-induced cases. Only 4 (2.2%) cases were attributed to sildenafil or tadalafil use. Although 15.1% were classified as idiopathic priapism, drug use (marijuana, cocaine, methamphetamine) was found in 25% of idiopathic cases.

### Diagnostics and interventions

35.9% of priapism presentations in the emergency department included a CBG test, while 9.2% had a penile ultrasound. In outpatient clinic visits, only 2.0% underwent a CBG test, and 6.0% received a penile ultrasound. Most cases required intervention (92.5%), with only 7.5% self-resolving. Of the 131 cases that presented to the emergency department, urology was consulted in 102 (77.8%) cases. No cases were found to have undergone a proximal shunt of any kind. Diagnostics and interventions for the four most common etiologies can be found in Supplementary Table 1.

#### Follow-up and erectile dysfunction outcomes

**Fig. 3:**
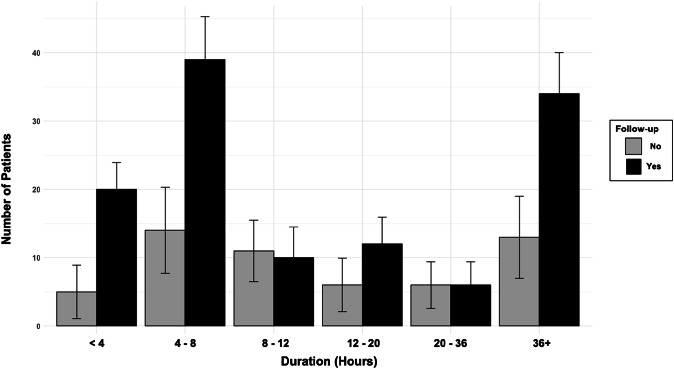
Urology follow-up by Priapism duration (hours) with confidence intervals.

Most patients (68.8%) followed up with a urologist after their priapism event. Patients with episodes lasting <4 h, 4–8 h, or >36 h were more likely to seek follow-up than those with durations of 8–36 h (Fig. 3). A point-biserial correlation showed a weak positive association (r = 0.025) between episode duration and follow-up likelihood.

In terms of insurance status, a Cramér’s V analysis showed a moderate-to-strong association between insurance type and follow-up status, with a value of V = 0. 373. Commercial payers and Medicare users had the highest follow-up rates with a urologist, at 78.6 and 75.8%, respectively. These rates were significantly higher compared to those with Medical/Medicaid insurance, where only 37.8% followed up with a urologist (*p* < 0.001). Only one of the four self-payer patients followed up with a urologist at our institution.

Of the 128 patients who followed up with a urologist, 67 (52.3%) had a history of ED, while 61 (47.7%) did not. Among those without a prior ED diagnosis, 47 (77.0%) were diagnosed with ED during subsequent follow-up visits. Of these newly diagnosed patients, 68.1% opted for oral phosphodiesterase-5 inhibitors (PDE5i) therapy, 4.3% chose ICI therapy, and 25.5% pursued inflatable penile prosthesis. A Sankey diagram exploring ED development and treatment outcomes stratified by priapism duration can be found in Supplementaary Fig. 1.

A multivariate logistic regression model was created to examine the factors influencing the likelihood of developing de novo ED after ischemic priapism (Table 4). In this model, the duration of ischemic priapism was found to be the most significant predictor. Patients with durations of 20–36 h (OR = 25.2, *p* = 0.007) and 36+ hours (OR = 61.3, *p* < 0.001) had markedly higher odds of developing ED compared to those with a duration of 0–4 h. In contrast, duration categories of 4–8, 8–12, and 12–20 h showed higher odds of ED but lacked statistical significance. Neither age, diabetes, hypertension, hyperlipidemia, race, nor ethnicity were found to significantly impact the likelihood of developing ED after ischemic priapism (*p* > 0.05) (Table 4).

**Table 4:** Odds ratios, confidence intervals, and *p*-values from multivariate logistic regression predicting erectile dysfunction after priapism.

Variable	Odds Ratio (OR)	95% CI	*p*-value
Duration (hours)			
0–4 (Intercept) (*n* = 25)	0.35	0.03–2.85	0.357
4–8 (*n* = 53)	1.91	0.34–15.40	0.487
8–12 (*n* = 21)	1.90	0.20–18.60	0.558
12–20 (*n* = 18)	2.87	0.40–25.80	0.300
20–36 (*n* = 12)	25.20	2.79–355.00	**0.007**
36+(*n* = 47)	61.30	11.80–530.00	**<0.001**
Age	0.97	0.93–1.01	0.203
Hypertension	0.82	0.21–2.96	0.765
Diabetes	1.48	0.25–7.97	0.656
Hyperlipidemia	0.54	0.13–2.19	0.387

**Statistical analysis:** Multivariate analysis evaluated the impact of ischemic priapism duration, age, and comorbidities on ED outcomes, with patients grouped by duration (0–4, 4–8, 8–12, 12–20, 20–36, 36+ hours). The 0–4 h group served as the reference for ED risk. All analyses were performed in R (Version 4.4.1), with statistical significance defined as *p* < 0.05.

*CI* confidence interval.

## Discussion

This retrospective study at a high-volume institution identified 186 priapism cases over nine years, with an increasing trend, particularly among high-risk populations using ICI and certain medications. Even though CBG testing is a clinical principle recommended by AUA guidelines to differentiate between high- and low-flow priapism [1], it was rarely performed — and when it was, it did not seem to influence treatment outcomes. With over 96% of cases being ischemic priapism, the clinical presentation alone was sufficient to guide management. Therefore, providers should prioritize timely intervention, also in line with current AUA guidelines [1].

Additionally, our study found significant racial and socioeconomic disparities in priapism presentations. Black and Asian patients presented at younger ages than White patients, with Black patients also experiencing longer-duration priapism. Additionally, patients with Medi-Cal insurance presented younger and had longer median priapism durations compared to those with commercial insurance. This is the first study to highlight such significant differences in priapism duration and patient age across racial and insurance groups. Although limited research exists on socioeconomic disparities in priapism risk, one 2022 study found that Hispanic ethnicity, lower income, sickle-cell disease, and illicit drug use were associated with an increased risk of recurrent episodes [10]. Further research into priapism socioeconomic risk factors is essential to develop targeted interventions and educational strategies for high-risk populations.

Regarding etiologies, ICI therapy accounted for 54.8% of all priapism cases observed at our single institution, with at-home ICI users experiencing the highest rates of recurrent ischemic priapism. Patient errors and inadequate training may contribute to this risk, as existing literature suggests that 42% of ICI users make at least one error during administration [11]. Recreational ICI users were also at high risk, with long median priapism durations, consistent with recent literature in a similar metropolitan area [8]. Future research and public health initiatives should focus on educating ICI users about proper administration techniques and the dangers of using ICI without a prescription.

Medications, particularly trazodone, accounted for a significant portion of priapism cases in this study. While the historical incidence of priapism with trazodone use is low, recent literature suggests that the risk may be as high as 8% among trazodone users [12, 13]. Interestingly, current research has found that many patients prescribed trazodone are neither screened for a history of priapism nor informed about the potential risk of priapism, despite prior episodes being a known risk factor [12, 14]. Similarly, antipsychotics like quetiapine were common inducers of priapism, accounting for 11.9% of all medication-induced priapism episodes. Recent literature suggests that due to their α1-receptor-antagonist properties, antipsychotics place patients at higher risk for priapism, particularly those with a previous history [14]. Apart from psychiatric medications, PDE5i-induced priapism concerns many non-urologist providers and may prevent them from prescribing necessary medications. However, it remains a rare occurrence, as seen in this study, with current research indicating an incidence of around 0.4–2.9% [13, 15]. Ultimately, medication-induced priapism continues to account for a substantial proportion of priapism etiologies, underscoring the need for providers to conduct thorough medication reconciliations and closely monitor those with a history of priapism.

While follow-up rates were low and may skew our findings, we found that a significant proportion of ED-naïve men (77%) developed de novo ED following their ischemic priapism, particularly those with longer-durations. While the majority (68.1%) opted for oral PDE5i therapy, 25.5% sought surgical treatment through penile prosthesis implantation. Although PDE5i therapy is a common initial treatment modality for ED [16], studies have shown that erectile function may not improve with PDE5i treatment in patients with post-priapism ED [17]. Therefore, those with extended ischemic priapism may benefit most from surgical intervention. Recent studies have suggested that early penile prosthesis placement (<30 days) can result in lower complication rates, reduced fibrosis, and may even improve patient satisfaction for prolonged ischemic priapism [18–20]. Given the high demand for ED treatments following ischemic priapism, healthcare providers must be well-informed about the available options, especially for patients where the return of erectile function is unlikely. Additionally, follow-up care is essential and should be emphasized for patients with extended ischemic priapism, as timely intervention can improve sexual outcomes and decrease long-term complications.

Our multivariate logistic regression supports that ischemic priapism duration is a strong predictor of ED development. This is consistent with AUA guidelines recommending that providers counsel patients that the likelihood of erectile function recovery is low after events lasting 36 h or longer [1]. Consistent with existing guidelines, patients with ischemic priapism episodes lasting between 20 and 36 h had a much higher odds ratio of developing ED (OR 25.2, *p* = 0.007) compared to those whose priapism lasted <4 h. Unsurprisingly, this was even higher in those with ischemic priapism lasting over 36 h (OR = 61.3, *p* < 0.001). Our findings suggest that the risk of irrecoverable erectile function may increase before 36 h, building on prior studies showing irreversible penile tissue injury after 6 h [21]. However, given limited sample sizes, our data did not hold enough power when stratifying durations to find a more precise threshold. While age and other known comorbidities were not found to increase the likelihood of ED, the younger age and lower incidence of metabolic risk factors in this cohort may explain the lack of association with traditional ED risk factors.

Interestingly, 431 unique patients with ICD-10 codes for priapism (N48.3) were discovered during data extraction, however, only 186 of these cases were classified as true acute, recurrent, or non-ischemic priapism. The majority of cases were excluded due to other etiologies of penile pain and painful erections such as, but not limited to, Peyronie’s disease, infection, and nocturnal erections. A comprehensive chart review found that many of these misclassifications were primarily made by PCPs and non-urology providers, reflecting diagnostic differences across specialties. At the start of our study period, the transition from ICD-9 to ICD-10 codes may have influenced diagnostic patterns [22]. However, recent literature indicates that ICD-10 coding remains highly inaccurate, with error rates reaching up to 50% [23]. This underscores the need for PCPs and non-urology providers to familiarize themselves with urology-related ICD-10 codes to improve patient referrals and management.

Despite a robust cohort size and comprehensive data collection, our retrospective study has several limitations. One key limitation was the limited data available in the EMR. Due to the emergent nature of ischemic priapism, it is likely that many patients presented to our emergency department but received follow-up care at other institutions or were lost to follow-up. This limited our analysis of comorbidities and long-term follow-up data. Additionally, for patients classified as recurrent ischemic priapism, only the first presentation within the study period was analyzed, which may have skewed the findings, given that subsequent episodes may have had different etiologies, severities, and interventions. Moreover, we were unable to assess the true incidence of priapism as we did not have data on all emergency department presentations at our institution. Lastly, we acknowledge that our experience at a single institution may not be representative of other metropolitan areas or rural communities.

## Conclusion

Significant racial and socioeconomic disparities in priapism presentation were found in this retrospective cohort, with Black and Asian patients presenting younger and Black patients experiencing longer priapism episodes than White patients. Similarly, Medi-Cal/Medicaid patients also had prolonged episodes and lower follow-up rates. CBG testing was rarely used and did not impact treatment, reinforcing the need for prompt intervention based on clinical presentation alone. Major risk factors included ICI therapy and medication-induced priapism from trazodone and antipsychotics, emphasizing the need for better patient education and medication reconciliation. Among ED-naïve men, 77% developed de novo ED after ischemic priapism, with longer episode duration being the strongest predictor. Notably, ED risk rose sharply beyond 20 h. Further research will refine duration thresholds and explore factors driving low follow-up and ED development.

## Supplementary information

Supplemental Figure 1

Supplemental Table 1

Supplemental Figures/Tables

## Data Availability

The code and database used in this study are available upon request from the authors.
