# Supplementary material for: Risk factors, diagnosis, and long-term erectile dysfunction outcomes in priapism: a retrospective analysis of 186 cases from a single institution
Source: Int J Impot Res. 2025 Apr 22;38(1):23–9. doi: 10.1038/s41443-025-01076-9 (PMC12864028; doi:10.1038/s41443-025-01076-9)
Supplement: Supplementary file 1 — Supplemental Figure 1 [file 41443_2025_1076_MOESM1_ESM.docx]

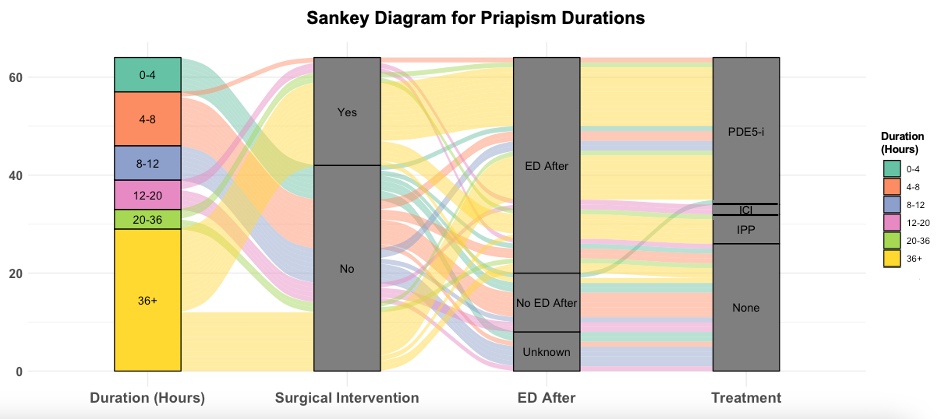


**Abbreviations**: ED: Erectile Dysfunction, PDE5-i: Phosphodiesterase 5 inhibitor; ICI: Intracavernosal Injection; IPP: Inflatable Penile Prothesis
