# Supplementary material for: Risk factors, diagnosis, and long-term erectile dysfunction outcomes in priapism: a retrospective analysis of 186 cases from a single institution
Source: Int J Impot Res. 2025 Apr 22;38(1):23–9. doi: 10.1038/s41443-025-01076-9 (PMC12864028; doi:10.1038/s41443-025-01076-9)
Supplement: Supplementary file 2 — Supplemental Table 1 [file 41443_2025_1076_MOESM2_ESM.docx]

|  | **Overall Presentations**  **(n=186)** | **ICI Therapy (n=102)** | **Medication**  **(n=42)** | **Idiopathic**  **(n=28)** | **Sickle Cell Disease (n=7)** |
| --- | --- | --- | --- | --- | --- |
| **Diagnostics Performed (%)** | | | |  |  |
| Corporal Blood Gas | 28.5 | 19.6 | 45.2 | 39.3 | 0 |
| Penile Ultrasound | 8.1 | 3.9 | 4.8 | 21.4 | 0 |
| **Interventions Performed (Medication and Surgical) (%)** | | | |  |  |
| Self-resolution (no intervention) | 7.5 | 5.9 | 14.3 | 3.6 | 14.3 |
| PO Terbutaline | 10.2 | 4.9 | 7.1 | 35.7 | 0 |
| Intracavernosal Phenylephrine | 81.2 | 87.3 | 73.8 | 82.1 | 71.4 |
| Corporal Aspiration and Irrigation | 72.6 | 75.5 | 69.0 | 82.1 | 42.9 |
| Distal Corporoglanular Shunt (any type) | 19.9 | 10.8 | 28.6 | 32.1 | 28.6 |
| Corporal Tunneling | 5.4 | 3.9 | 9.5 | 3.6 | 0 |
