# Supplementary material for: Risk factors, diagnosis, and long-term erectile dysfunction outcomes in priapism: a retrospective analysis of 186 cases from a single institution
Source: Int J Impot Res. 2025 Apr 22;38(1):23–9. doi: 10.1038/s41443-025-01076-9 (PMC12864028; doi:10.1038/s41443-025-01076-9)
Supplement: Supplementary file 3 — Supplemental Figures/Tables [file 41443_2025_1076_MOESM3_ESM.docx]

**Supplemental Figures/Tables**

**Supplemental Figure 1:** Sankey Diagram for Priapism Durations (Hours) with ED outcomes and treatment.

**Supplemental Table 1:** Diagnostic and Intervention Information by the 4 most common priapism etiologies.
